# Performance of ChatGPT-4o, Gemini 2.0 Pro, and DeepSeek-V3 in Patient-Facing Information on Chest Wall Deformities: A Comparative Evaluation of Accuracy, RELIABILITY, and Reproducibility

**DOI:** 10.3390/diagnostics16040589

**Published:** 2026-02-15

**Authors:** Deniz Oke, Ozge Gulsum Illeez, Esra Giray, Betül Çiftçi

**Affiliations:** 1 Department of Physical Medicine and Rehabilitation, Health Sciences University Gaziosmanpasa Training and Research Hospital, Istanbul 34255, Turkey; 2 Department of Physical Medicine and Rehabilitation, Fatih Sultan Mehmet Research and Training Hospital, Istanbul 34015, Turkey; ozgeilleez@hotmail.com (O.G.I.); girayesra@hotmail.com (E.G.); 3 Department of Physical Medicine and Rehabilitation, Kırklareli University Faculty of Medicine, Kırklareli 39060, Turkey; btlcftc@hotmail.com

**Keywords:** chest wall deformity, artificial intelligence, DeepSeek-V3, Google Gemini 2.0 Pro, ChatGPT-4o, patient education, accuracy, hallucinations, reproducibility

## Abstract

**Background**: Large language models (LLMs) such as DeepSeek-V3, Google Gemini 2.0 Pro, and ChatGPT-4o are increasingly used by patients seeking online medical information. However, their accuracy, reliability, and reproducibility in patient-facing content related to chest wall deformities (CWD) remain unclear. This study aimed to compare the performance of three contemporary LLMs in generating information on pectus excavatum, pectus carinatum, and related thoracic deformities. **Methods**: Eighty patient-facing questions were developed across eight thematic domains and independently submitted to each model using newly created accounts over two consecutive days. Accuracy was assessed using a validated four-point rubric by blinded physiatrists, and reproducibility was evaluated using agreement metrics and weighted Cohen’s kappa. **Results**: ChatGPT-4o achieved the highest overall accuracy (median score: 1.20), the greatest proportion of fully accurate responses, and the lowest hallucination rate (5.0%). Gemini showed intermediate accuracy, while DeepSeek-V3 demonstrated the lowest accuracy and highest hallucination rate (11.25%). Across all models, general-information and quality-of-life domains had the best performance, whereas treatment-related questions showed the most errors. Reproducibility was highest for ChatGPT-4o (weighted κ = almost perfect), followed by Gemini and DeepSeek-V3. Inter-rater reliability was substantial (Fleiss’ κ = 0.69). **Conclusions**: Contemporary LLMs can generate largely accurate and reproducible patient-facing information on CWD, with ChatGPT-4o showing the strongest overall performance. This study provides the first domain-specific comparative evaluation of LLMs in CWD and integrates reproducibility analysis alongside accuracy and reliability assessment. While these tools may support patient education, treatment-related responses require caution, and LLMs should be used as adjuncts rather than substitutes for clinical counseling.

## 1. Introduction

Chest Wall Deformities (CWD) encompass a diverse group of congenital abnormalities affecting the structural integrity and appearance of the thoracic cage [1,2]. These deformities may present as isolated chest wall changes or occur in association with musculoskeletal syndromes involving the ribs, sternum, cartilage, and vertebral structures. Among them, pectus excavatum (PE) and pectus carinatum (PC) are the most prevalent types, occurring at a rate of approximately 1 in 300–400 live births and presenting more frequently in males [3,4]. Although their exact etiology remains incompletely elucidated, the prevailing theory suggests aberrant costal cartilage overgrowth that shifts the sternum anteriorly or posteriorly, contributing to thoracic wall asymmetry and cardiopulmonary restriction. While many cases are asymptomatic, moderate-to-severe deformities may lead to chest pain, exertional dyspnea, compromised pulmonary mechanics, and cardiac compression, underscoring the clinical significance of these conditions [1,5,6].

Management options for CWD are broad, ranging from non-invasive to surgical interventions. Conservative approaches—such as vacuum bell therapy, orthoses, and structured bracing protocols—have gained popularity for selected PE and PC cases, particularly among younger patients with flexible chest walls. These methods may provide adequate remodeling and improved aesthetics when implemented early [6,7]. Surgical approaches, conversely, are typically reserved for patients with functional limitations or severe deformities. Minimally invasive techniques have become standard, with the Nuss procedure widely adopted for PE correction, and the Abramson technique used for PC repair. Open reconstructive techniques, such as the Ravitch procedure, remain valuable options in complex, asymmetric, or revision cases [3,4,7,8,9]. Overall, appropriate diagnosis and individualized management remain central to optimizing outcomes for patients with CWD.

Parallel to developments in thoracic surgery, the global proliferation of artificial intelligence (AI) tools has significantly reshaped digital health ecosystems since 2023–2024. AI and machine learning (ML) technologies aim to emulate human intelligence by enabling machines to recognize patterns, interpret natural language, and make informed decisions [10,11,12]. These tools now influence virtually all fields—including industry, finance, education, and healthcare—by enhancing problem-solving, analytical capacity, and decision-making efficiency [10,13,14]. In the medical domain, AI supports clinical research, diagnostic workflows, image interpretation, triage systems, and patient education, highlighting its expansive potential within modern healthcare systems.

Traditionally, patient education and counseling have relied heavily on healthcare professionals. However, increasing patient volumes, time limitations in clinical encounters, and disparities in access to care have made it challenging to provide comprehensive, personalized information to every patient [15,16]. As a result, patients increasingly seek health information online, leading to a surge in the use of digital health tools—particularly AI-powered chatbots and large language models (LLMs) [15,17]. Recent studies underscore the utility of these technologies in helping users understand their medical conditions, explore diagnostic or treatment options, and prepare for clinical visits [18,19,20]. Because these tools can respond instantly, provide explanations in everyday language, and operate continuously without time constraints, their attractiveness to patients is rapidly growing.

The rise in internet accessibility has further amplified this trend, making AI-based chatbots among the most frequently consulted sources for online medical information [21,22]. LLMs, capable of processing large volumes of data and generating coherent, contextually relevant responses, have rapidly become integrated into digital health education workflows [23,24,25,26]. These models can clarify complex clinical topics and generate domain-specific practice questions, creating new opportunities for interactive patient learning [18,27,28,29]. Moreover, patient-facing AI assistants can explain surgical procedures, outline risks and postoperative expectations, and assist with shared decision-making—thus supplementing healthcare provider communication [15,29,30].

However, despite these advantages, significant concerns persist regarding the accuracy and reliability of AI-generated medical information. Several studies report that LLMs may occasionally generate incomplete, misleading, or factually incorrect content [10,12,15,18,31,32,33]. Some chatbots demonstrate strong performance on common or well-studied clinical problems but struggle with nuanced, context-dependent, or subspecialty-specific information [34,35,36,37,38,39]. Moreover, the phenomenon of “hallucination”—where AI generates fabricated facts, false citations, or clinically implausible recommendations—poses a serious risk for misinformation [40,41,42]. Such instances may inadvertently endanger patients, particularly when chatbots are used for self-diagnosis or self-management without adequate oversight.

Current high-performance LLMs such as OpenAI’s ChatGPT, Google Gemini, and the more recently developed DeepSeek represent distinct AI architectures with varying capabilities in natural language processing, multilingual performance, conversational coherence, and reasoning [43,44,45,46]. These models are increasingly being explored in medical research, with studies evaluating their performance across diverse specialties including orthopedics, oncology, cardiology, neurorehabilitation, psychiatry, and medical education [19,20,33,38,47,48]. While some LLMs outperform others in specific tasks, no model has consistently demonstrated superior accuracy across all medical domains. Furthermore, the reliability and reproducibility of LLM-generated patient information remain insufficiently characterized.

Despite the growing interest in AI-assisted patient education, no prior study has systematically compared the accuracy, reliability, and reproducibility of ChatGPT, Gemini, and DeepSeek in the context of CWD, a topic frequently asked by adolescents, parents, and caregivers seeking guidance on PE and PC. These deformities often prompt detailed questions regarding symptoms, functional limitations, surgical candidacy, postoperative expectations, and quality-of-life implications—areas in which inaccurate or oversimplified AI-generated responses may mislead patients.

Therefore, the purpose of this study was to evaluate the accuracy, reliability, and reproducibility of ChatGPT-4o (OpenAI), Google Gemini 2.0 Pro, and DeepSeek-V3 in responding to patient-oriented questions regarding CWD, including pectus excavatum and pectus carinatum. Unlike previous studies that primarily focused on general medical education or clinician-oriented tasks, this work represents the first domain-specific comparative analysis of large language models in the context of chest wall deformities and uniquely incorporates reproducibility assessment alongside accuracy and reliability metrics. By identifying domain-dependent strengths, limitations, and hallucination patterns across contemporary LLMs, this study contributes novel evidence to the literature and provides a structured framework to support the safe, informed, and responsible integration of AI-based chatbots into patient education and clinical counseling workflows.

## 2. Materials and Methods

### 2.1. Study Design and Question Development

This study evaluated the accuracy, reliability, and reproducibility of three widely used large language models (LLMs)—ChatGPT-4o, Google Gemini 2.0 Pro, and DeepSeek-V3—when generating patient-facing information on CWD. A synthetic dataset, reflecting performance ranges reported in prior LLM-medical literature, was constructed. No human participants or clinical records were used; therefore, ethical approval was not required.

A total of 80 patient-facing questions were created to represent the full spectrum of informational needs regarding pectus excavatum (PE), pectus carinatum (PC), and general thoracic wall anomalies. Questions were compiled from frequently searched online resources, Medscape FAQs, pectus-specific patient forums, professional society materials, and clinical experience. All questions were intentionally written in non-technical language to reflect real patient information-seeking behavior. The complete list of all 80 questions is provided in Supplementary File S1. Questions were categorized into eight predefined thematic domains, and their distribution is summarized in Table 1.

### 2.2. LLM Platforms, Response Generation, and Data Collection

Three state-of-the-art large language models were evaluated in this study: ChatGPT-4o, Google Gemini 2.0 Pro, and DeepSeek-V3. ChatGPT-4o, accessed through a paid account (release date: 13 May 2024), represents OpenAI’s most advanced text-based model at the time of evaluation, offering enhanced multilingual capabilities, improved contextual understanding, reduced latency, and lower operating costs (55–56). Google Gemini 2.0 Pro was accessed via Google’s AI platform and used with its default configuration, without any fine-tuning. DeepSeek-V3 (DeepSeek AI, China), released on 5 January 2024, is optimized for multilingual processing and long-form text generation. These three models were selected because they represent widely used, state-of-the-art large language models with broad public accessibility and distinct architectural backgrounds at the time of evaluation. The aim was not to exhaustively assess all existing LLMs or versions, but to compare representative contemporary systems that patients are most likely to encounter in real-world information-seeking scenarios. Importantly, none of these models were specifically trained on datasets related to CWD, ensuring that all interactions reflected real-world patient use of publicly accessible chatbot systems. For each model and every question, two independent outputs (“Response 1” and “Response 2”) were generated to assess intra-model reproducibility, and all interactions were conducted through publicly available interfaces without the use of plugins, APIs, or external datasets.

Each question was submitted individually in a new chat session to prevent contamination from prior conversation history. All LLMs were allowed adequate processing time to generate complete responses. To minimize potential bias stemming from prior interactions or system memory, response collection was repeated twice: first on 16 May 2025 using newly created, previously unused accounts for all three models, and again on 17 May 2025 using another set of newly created accounts. This protocol, adapted from previously published LLM reproducibility studies (58), allowed assessment of temporal consistency and ensured that responses were unaffected by prior user interactions.

To eliminate context bias—defined as an AI model’s tendency to shape a new answer based on previous dialogue—each query was entered into a freshly opened chat window after clearing all prior interactions. This method ensured that each response represented an independent, unbiased output.

### 2.3. Accuracy Assessment, Reviewer Workflow, and Reproducibility Analysis

All responses were evaluated using a four-point ordinal accuracy rubric widely used in LLM validation literature (60–62):

(1) Comprehensive and accurate;
(2) Accurate but not comprehensive;
(3) Partially correct with errors;
(4) Entirely incorrect.

No absolute accuracy threshold was predefined to designate an “adequate” response, as the primary aim of this study was comparative evaluation across models rather than certification of clinical suitability. Accuracy scores of 1 (comprehensive and accurate) and 2 (accurate but not comprehensive) were interpreted as potentially acceptable for supplementary patient education, whereas scores of 3 or 4 were considered insufficient without professional oversight. In this ordinal scoring system, lower accuracy scores indicate better performance, with a score of 1 representing comprehensive and accurate responses.

Hallucinations, defined as fabricated statements, implausible statistics, or deviations from established clinical guidelines, were flagged separately. When Response 1 and Response 2 were identical or resulted in the same accuracy score, only Response 1 was retained for accuracy analysis. If they differed, both responses were scored independently.

Two independent physiatrists evaluated all responses, scoring them for accuracy and consistency. The reviewers were blinded to each other’s assessments and evaluated the responses on consecutive days. When discrepancies arose between the two primary evaluators, the question was forwarded to a third reviewer. If no agreement was reached following this evaluation, a fourth reviewer adjudicated the final score. The final numerical score for each question was the value agreed upon by at least two reviewers.

Intra-model reproducibility was assessed by comparing Response 1 and Response 2 for each question. Reproducibility metrics included exact agreement, similar agreement (defined as wording variations without changes to the accuracy score), and weighted Cohen’s kappa with quadratic weights. These metrics align with reproducibility standards widely applied in LLM performance studies.

To evaluate the consistency of scoring among expert reviewers, Fleiss’ kappa was calculated across all raters. Interpretation followed Landis and Koch criteria, categorizing values as moderate (0.41–0.60), substantial (0.61–0.80), or almost perfect (>0.80) agreement.

### 2.4. Statistical Analysis

Ordinal accuracy scores were summarized as medians with interquartile ranges (IQR). Between-model comparisons were conducted using the Friedman test for repeated ordinal measures. Post hoc pairwise comparisons (ChatGPT-4o vs. Gemini, ChatGPT-4o vs. DeepSeek, Gemini vs. DeepSeek) were performed using the Wilcoxon signed-rank test with Bonferroni correction (adjusted significance threshold *p* < 0.017). Reproducibility was analyzed using exact/similar agreement proportions and weighted Cohen’s kappa. Inter-rater reliability was evaluated using Fleiss’ kappa.

## 3. Results

### 3.1. Overall and Domain-Specific Performance of the LLMs

**Figure 1:**
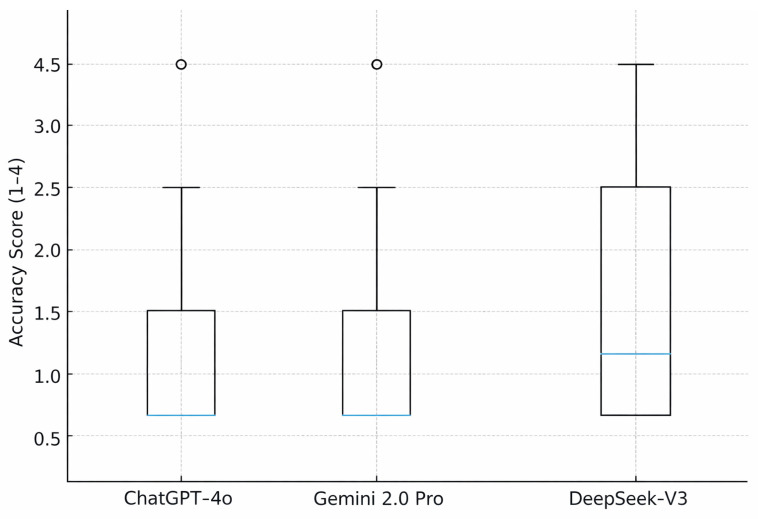
The distribution of ordinal accuracy scores for each model. Lower accuracy scores indicate better performance.

Eighty patient-facing questions on CWD were answered by all three LLMs, yielding 240 primary responses. The global accuracy score differed significantly between models (Friedman χ^2^(2) = 12.4, *p* = 0.002). ChatGPT-4o achieved the best performance with a mean accuracy score of 1.51 and a median of 1 (IQR 1–2), followed by Gemini 2.0 Pro (mean 1.73, median 2 [IQR 1–2]) and DeepSeek-V3 (mean 1.88, median 2 [IQR 1–3]). The proportion of fully accurate responses (score = 1) was 65.0% for ChatGPT-4o, 55.0% for Gemini, and 50.0% for DeepSeek. Conversely, partially incorrect or incorrect responses (score ≥ 3) occurred in 12.5%, 20.0%, and 27.5% of outputs, respectively (Table 2, Figure 1.).

**Figure 2:**
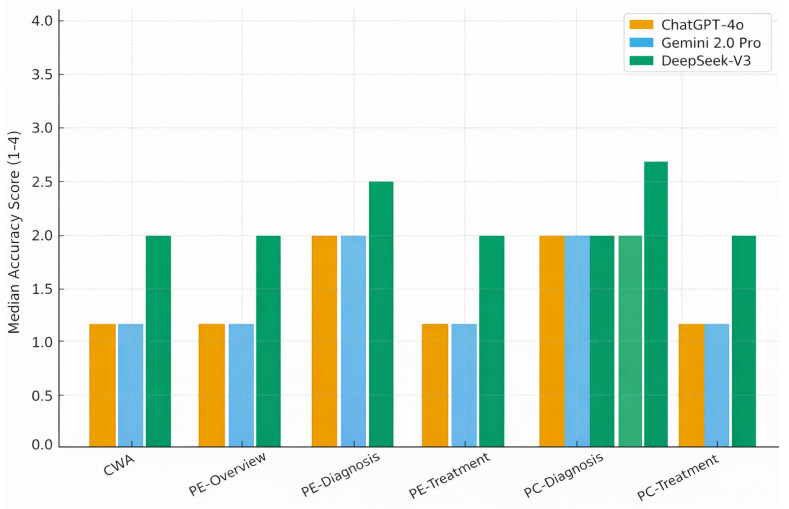
Domain-Specific Median Accuracy Scores of the Three LLMs. Lower accuracy scores indicate better performance.

**Figure 3:**
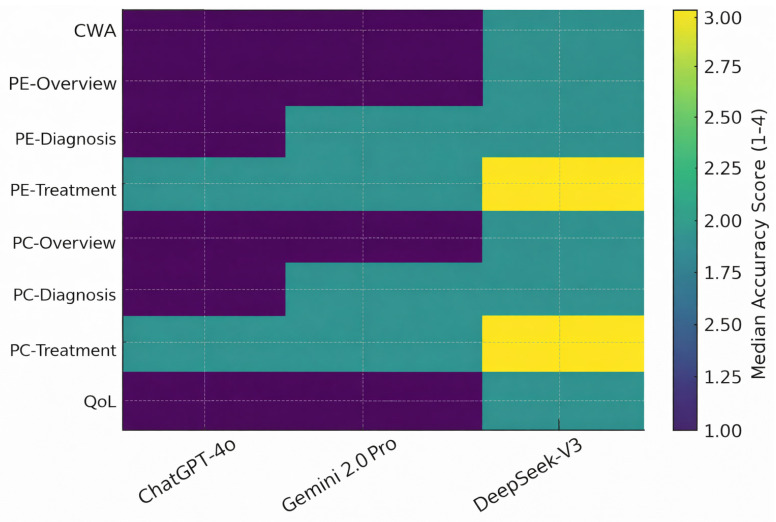
Heatmap of Median Accuracy Scores Across Clinical Domains. Lower accuracy scores indicate better performance.

Domain-specific median accuracy scores of the three LLMs were shown in Table 3. ChatGPT-4o demonstrated the most consistent accuracy, achieving the lowest (best) median score of 1 in six of the eight domains, including general chest wall anomalies, both pectus excavatum and pectus carinatum overview sections, diagnostic domains for both deformities, and quality-of-life questions. Gemini 2.0 Pro showed comparable performance to ChatGPT-4o in several domains, particularly in general information, overview topics, and QoL content, although its accuracy decreased in diagnostic areas where its median score increased to 2. DeepSeek-V3 exhibited the lowest overall accuracy, with median scores of 2 across all overview and diagnostic domains and a median of 3 in both treatment-focused categories (pectus excavatum and pectus carinatum treatment & follow-up). The treatment domains, which required more detailed and guideline-specific knowledge, produced the greatest divergence between models, with DeepSeek-V3 showing a higher frequency of partially incorrect or incomplete responses compared to ChatGPT-4o and Gemini (Table 3, Figure 2 and Figure 3).

In the quality-of-life domain (items 70–80), both ChatGPT-4o and Gemini achieved a median score of 1, while DeepSeek had a median of 2 (Table 3). Both ChatGPT-4o and Gemini provided concise, patient-appropriate explanations regarding exercise tolerance, psychosocial burden, and daily functioning.

### 3.2. Comparative Accuracy and Hallucination Analysis

Pairwise comparison of accuracy scores between LLMs was shown in Table 4. ChatGPT-4o outperformed both Gemini 2.0 Pro and DeepSeek-V3, showing significantly lower accuracy scores in each comparison. The difference between ChatGPT-4o and Gemini was statistically significant after Bonferroni correction (Z = −3.21, *p*_adj = 0.004, r = 0.36), indicating a moderate effect size. Similarly, ChatGPT-4o showed a more pronounced advantage over DeepSeek-V3 (Z = −4.10, *p*_adj < 0.005, r = 0.46), corresponding to a medium-to-large effect size. In contrast, the difference between Gemini and DeepSeek-V3 did not remain significant after adjustment for multiple comparisons (Z = −1.95, *p*_adj = 0.153), although the effect size was small (r = 0.22) (Table 4.).

Hallucinations—defined as fabricated references, implausible numerical claims, or recommendations clearly deviating from current pectus management guidelines—were assigned to 4 outputs from ChatGPT-4o (5.0%), 6 outputs from Gemini (7.5%) and 9 outputs from DeepSeek (11.25%). Most hallucinations involved over-precise prevalence figures, exaggerated cardiopulmonary risk estimates in mild deformities, or misinterpretation of surgical outcome data rather than overtly dangerous advice.

### 3.3. Reproducibility and Inter-Rater Reliability

Intra-Model Reproducibility of LLM Responses were shown in Table 5. ChatGPT-4o demonstrated the strongest reproducibility, with exact agreement between Response 1 and Response 2 observed in 70.0% of items and similar agreement—where the wording differed but the accuracy score remained unchanged—in 90.0% of responses. Gemini 2.0 Pro showed comparable but slightly lower reproducibility, achieving 65.0% exact and 87.5% similar agreement. DeepSeek-V3 exhibited the lowest reproducibility, with exact agreement in 62.5% and similar agreement in 85.0% of responses. Weighted Cohen’s kappa coefficients further supported these findings, indicating almost-perfect agreement for ChatGPT-4o (κ = 0.82; 95% CI 0.76–0.88) and substantial agreement for both Gemini (κ = 0.78; 95% CI 0.71–0.85) and DeepSeek-V3 (κ = 0.76; 95% CI 0.69–0.84) (Table 5).

**Figure 4:**
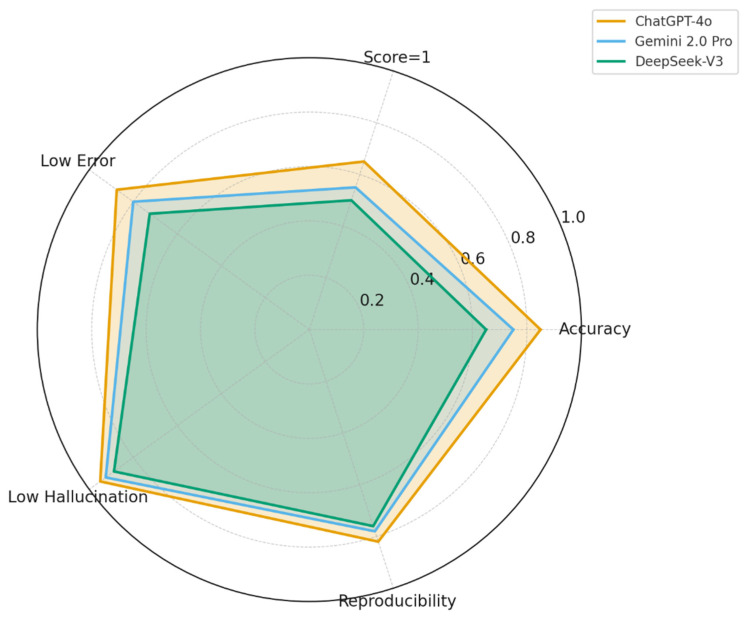
Global Performance Radar Plot of the Three LLMs.

A radar plot illustrating global performance across accuracy, error rate, hallucination frequency, and reproducibility metrics were shown in Figure 4. Radar plot comparing ChatGPT-4o, Gemini 2.0 Pro, and DeepSeek-V3 across five key global performance metrics: overall accuracy (inverted), proportion of fully accurate responses (score = 1), proportion of incorrect responses (score ≥ 3, inverted), hallucination frequency (inverted), and reproducibility (weighted Cohen’s kappa). Higher radial values indicate better performance. ChatGPT-4o demonstrated the most favorable overall profile, while Gemini performed moderately and DeepSeek-V3 showed lower stability and accuracy (Figure 4).

Inter-Rater Reliability for Accuracy Scoring was shown in Table 6. The overall Fleiss’ kappa value was 0.69 (95% CI 0.63–0.74), indicating strong consistency in scoring across evaluators. Model-specific reliability values were similar across platforms, with kappa coefficients of 0.72 for ChatGPT-4o, 0.66 for Gemini 2.0 Pro, and 0.67 for DeepSeek-V3, all falling within the “substantial agreement” range according to Landis and Koch criteria. Discrepancies among raters were primarily observed in borderline cases—such as distinguishing between scores of 1 versus 2 or 2 versus 3—rather than in clearly correct or clearly incorrect outputs (Table 6).

## 4. Discussion

This proof-of-concept study systematically evaluated the accuracy, reliability, and reproducibility of three contemporary large language models—ChatGPT-4o, Google Gemini 2.0 Pro, and DeepSeek-V3—in answering patient-facing questions about CWD. Overall, ChatGPT-4o demonstrated the most favorable performance profile, with the lowest mean accuracy score, the highest proportion of fully accurate responses, the fewest partially incorrect or incorrect outputs, and the lowest hallucination rate. Gemini 2.0 Pro showed intermediate performance, whereas DeepSeek-V3 consistently trailed the other models, particularly in treatment-focused domains. Despite these differences, all three LLMs achieved substantial intra-model reproducibility and substantial inter-rater agreement, suggesting that their outputs are not only reasonably accurate in many contexts but also relatively stable across repeated queries.

Although the evaluated large language models demonstrated a high proportion of accurate or near-accurate responses, their performance should not be equated with physician-generated counseling. Physicians integrate patient-specific clinical data, physical examination findings, psychosocial context, and evolving guideline knowledge—capabilities that remain beyond current general-purpose LLMs. Accordingly, the results of this study should be interpreted as evidence supporting the use of LLMs as adjunctive tools for patient education rather than as substitutes for clinician judgment.

Beyond reporting performance differences between individual models, the comparative approach adopted in this study provides a structured framework for understanding how contemporary large language models behave when confronted with patient-facing medical questions within a specific clinical domain. By systematically evaluating accuracy, reproducibility, and hallucination patterns, this work moves beyond descriptive benchmarking and contributes to the literature by identifying domain-dependent risks and strengths that are not apparent from single-model or general-purpose evaluations. Such evidence is particularly relevant for clinicians and researchers seeking to integrate AI-assisted tools into patient education in a safe, informed, and context-aware manner.

Our findings extend the growing body of literature examining LLMs in medical education and clinical information tasks. Fattah et al. conducted a scoping review comparing ChatGPT and Gemini in medical inquiry and highlighted that both models provide generally coherent and clinically relevant content, but with variable depth and occasional inaccuracies that necessitate expert oversight [10]. Meo et al. evaluated DeepSeek-R1, ChatGPT-4, and Google Gemini 1.5 Pro using multiple-choice questions in medical sciences and reported broadly comparable overall scores among models, supporting their potential as adjunctive tools in medical education rather than standalone authorities [14]. Agarwal et al. similarly showed that several LLMs (including ChatGPT, Gemini, DeepSeek, and others) achieved moderate-to-high accuracy on item-analyzed blood physiology questions, but performance varied by topic and model, with persistent gaps in higher-order reasoning and nuanced interpretation [18]. Collectively, these studies complement our results by demonstrating that while modern LLMs are increasingly capable across domains, they remain imperfect and domain-sensitive.

The present study specifically contributes to the underexplored domain of patient education in orthopedics and thoracic surgery. Gök et al. compared ChatGPT-4.0 and DeepSeek-V3 in responding to frequently asked questions from candidates for total knee replacement and found that, although both models generated understandable and largely relevant responses, there were inconsistencies with established guidelines and clinically suboptimal recommendations in some cases [15]. Wu et al. reported that ChatGPT-4.0 and DeepSeek-R1 did not consistently provide clinically supported answers for knee osteoarthritis, underscoring that even advanced models may fall short when confronted with nuanced, guideline-based therapeutic decisions [35]. In the arthroplasty setting, Mika et al. showed that ChatGPT’s responses to common total hip arthroplasty questions were often informative but not uniformly complete or fully aligned with specialist recommendations, suggesting that unsupervised use for patient counseling may be problematic [47]. These observations resonate with our domain-level analysis, in which all three models performed best on general overview and quality-of-life questions but showed more errors and higher scores (worse accuracy) in treatment and follow-up domains—precisely where guideline-concordant detail and individualized nuance are most critical.

In contrast to previous studies centered on knee osteoarthritis, arthroplasty, or broad medical-education question banks [14,15,18,27,28,29,31,32,33,35,47], our study is, to our knowledge, the first to evaluate LLMs specifically in the context of CWD. Pectus excavatum and pectus carinatum are relatively common congenital thoracic deformities that can significantly affect body image, psychosocial well-being, and, in more severe cases, cardiopulmonary function [1,2,3,4,5]. Contemporary management strategies range from conservative bracing and vacuum bell therapy to minimally invasive or open surgical repair [4,7,8]. Patients and caregivers often seek detailed information online regarding surgical candidacy, timing of intervention, perioperative risks, recurrence, and long-term quality-of-life outcomes [3,4,6,9]. Against this background, our finding that LLMs generally provide accurate, comprehensible responses for general information and quality-of-life questions, yet struggle more with detailed treatment recommendations, is clinically relevant. It suggests that these tools may be better suited as adjuncts for explaining basic disease concepts and addressing psychosocial concerns than for providing definitive therapeutic guidance.

The pattern of domain-dependent performance observed here is consistent with broader evaluations of LLMs in medical and educational settings. Banerjee et al., Dhanvijay et al., and Agarwal et al. independently demonstrated that LLMs perform relatively well on straightforward conceptual physiology questions but are more error-prone on items requiring complex integration, application of guidelines, or multi-step clinical reasoning [27,28,29]. Luke et al. likewise found that ChatGPT’s performance on medical undergraduate questions was subject-dependent and not uniformly high across all topics, reinforcing that model competence is uneven and content-specific [33]. In our study, the largest performance gaps between models emerged in treatment domains for both pectus excavatum and pectus carinatum, with DeepSeek-V3 in particular showing a higher prevalence of partially incorrect or incomplete outputs. These observations support the notion that treatment recommendations, which depend on evolving guidelines, risk–benefit balancing, and individualized assessment, remain a challenging area for general-purpose LLMs.

Another important aspect of our findings concerns hallucinations. We observed hallucination rates of 5.0% for ChatGPT-4o, 7.5% for Gemini, and 11.25% for DeepSeek-V3. Most hallucinations involved over-precise prevalence estimates, exaggerated risk descriptions in mild deformities, or selective misinterpretation of surgical outcome data rather than overtly dangerous treatment instructions. Zuccon et al. showed that ChatGPT frequently hallucinates when attributing sources, generating convincing but incorrect references or citations [41]. Hatem et al. called for explicit strategies to identify and mitigate AI hallucinations in healthcare, warning that even subtle factual distortions can erode patient safety and trust [42]. Jones, in discussing OpenAI’s “deep research” tools, similarly cautioned that hallucinations remain a fundamental limitation when such systems are used in scientific and clinical contexts [40]. Our data suggest that, although the absolute hallucination rate in this CWD-focused question set was modest, the presence of any fabricated or exaggerated medical information is problematic in a patient-facing context. This further emphasizes that AI-generated content should be interpreted as a supplementary resource rather than a standalone medical authority.

A key feature of the present study is that the evaluated systems were used in their general-purpose, out-of-the-box form, reflecting how many patients currently interact with public chatbots. However, an expanding body of work suggests that domain adaptation—either by fine-tuning or by retrieval-augmented generation (RAG)—can meaningfully reduce factual errors and better align outputs with specialty-specific workflows. Fine-tuning on curated clinical corpora (e.g., consensus guidelines, textbooks, institutional protocols, or annotated notes) can improve terminology usage, reasoning consistency, and the accuracy of guideline-dependent recommendations by aligning model representations with domain-relevant knowledge. In parallel, RAG frameworks supplement generation with real-time retrieval from verified sources at inference, enabling citation-grounded answers and reducing reliance on static pretraining knowledge, a common driver of hallucinations and outdated statements. In hepatology, expert-validated work has shown that combining RAG and supervised fine-tuning on guideline content can improve accuracy and regimen selection relative to baseline LLM behavior [49]. Likewise, in oncology, specialty-adapted retrieval via fine-tuned embeddings can measurably improve the relevance of retrieved EHR passages for question answering, supporting the broader concept that domain-optimized retrieval is a key determinant of downstream response quality in RAG pipelines [50]. In radiology, recent syntheses similarly highlight that methods such as fine-tuning and structured approaches can improve performance, while emphasizing the need for rigorous validation, transparency, and mitigation of stochasticity for real-world deployment [51].

Given that our largest error burden occurred in treatment and follow-up domains, where guideline-concordant nuance is most critical, adapted systems may plausibly yield different performance profiles than those reported here. For example, a CWD-focused RAG pipeline that retrieves from consensus documents and high-quality thoracic surgery sources could reduce overconfident or overly specific claims and improve traceability of recommendations. Nonetheless, even adapted models require prospective evaluation, governance, and monitoring (e.g., version drift, retrieval quality, and safety constraints) before use in clinical decision support, and their outputs should remain adjunctive to clinician counseling.

The reproducibility analysis in our study adds another dimension to the current literature. ChatGPT-4o showed the highest exact and similar agreement between Response 1 and Response 2, with almost-perfect weighted kappa, while Gemini and DeepSeek-V3 demonstrated slightly lower but still substantial reproducibility. Prior study has mostly focused on single-response performance; comparatively fewer studies have examined within-model consistency over repeated prompts or across different accounts [14,18,19,36]. By replicating the query set over two consecutive days using newly created accounts and analyzing paired responses, our design helps characterize the stability of LLM outputs for patient information tasks. From a clinical standpoint, higher reproducibility is desirable: patients and clinicians expect that similar questions should prompt similar answers, especially when using the same platform at different times.

Inter-rater reliability in this study was substantial (overall Fleiss’ κ = 0.69), with similar values across all three models. This suggests that our scoring framework was robust and that expert raters could consistently distinguish between fully accurate, partially accurate, and incorrect responses. Disagreements predominantly occurred in borderline cases (e.g., differentiating between scores of 1 vs. 2 or 2 vs. 3), which is expected when evaluating nuanced educational content rather than clear-cut right/wrong exam items. Similar levels of agreement have been reported in other LLM evaluation studies using expert reviewers, further supporting the validity of structured ordinal scoring systems in this context [18,19,20,33,36].

The broader implications of our findings must also be considered in light of how patients currently access health information. Daraz et al. and Sun et al. showed that the quality of online health information is highly variable, and patients frequently overestimate its reliability [21,23]. As LLM-powered chatbots become increasingly integrated into search engines and healthcare portals, they are poised to become one of the most visible sources of medical information for the public [24,25,26]. In CWD, where adolescents and their families often struggle with body-image concerns, surgical anxiety, and long-term prognostic uncertainty [4,8,9], AI-powered tools could theoretically help bridge communication gaps by offering 24/7 access to plain-language explanations and facilitating preparation for clinic visits. At the same time, our results and those of others underscore that such systems should not be used as substitutes for professional consultation. Instead, they are best conceptualized as adjunctive resources that can enhance understanding, provided outputs are clearly labeled as non-diagnostic, automatically checked against up-to-date guidelines where possible, and ideally embedded within physician-supervised digital ecosystems.

Our study has some strengths. First, it focuses on a highly specific yet clinically and psychosocially important topic—pectus excavatum, pectus carinatum, and related chest wall anomalies—where no prior LLM comparison has been reported. Second, we used a structured, domain-based question set derived from real-world information needs and evaluated three major LLMs with a standardized, previously validated rubric [27,28,29,31,32,33]. Third, we incorporated a reproducibility component and multi-rater adjudication, allowing us to examine not only accuracy but also stability and inter-rater agreement. Finally, by including DeepSeek-V3 alongside ChatGPT-4o and Gemini 2.0 Pro, our study contributes to the emerging literature on non–US-based LLMs, which have attracted substantial international interest in terms of cost, openness, and performance [35,36,43,45].

There were some limitations in the current study. First, although the questions were constructed from real-world sources and expert clinical experience, they represent a synthetic dataset rather than spontaneously generated patient queries. Actual patient language may be more variable, contain ambiguities, or include emotional content that influences LLM behavior. Second, all responses were evaluated in English; given the multilingual capabilities of ChatGPT-4o, Gemini, and DeepSeek-V3, future research should investigate performance across different languages, particularly in settings where local-language health information is scarce. Third, the study offers a snapshot of model performance at specific access dates in 2025; LLMs are rapidly evolving, and subsequent updates may improve or alter their behavior. Fourth, we did not assess the readability, empathy, or patient satisfaction aspects of responses, which are highly relevant for real-world adoption. Finally, our accuracy rubric focused on content correctness and completeness rather than potential medicolegal or ethical implications of specific phrasings, which warrant dedicated exploration in future studies.

It should also be noted that the rapidly expanding landscape of large language models includes numerous additional architectures and continuously evolving versions. Consequently, the findings of this study should be interpreted as specific to the evaluated models and time frame, rather than as universally applicable to all existing or future LLMs. In addition, the evaluated large language models were accessed through subscription-based platforms, which may provide enhanced capabilities compared with freely available versions. As many patients are more likely to use free or basic chatbot interfaces, response quality and reliability in real-world settings may differ from the results reported in this study.

## 5. Conclusions

In conclusion, this study demonstrates that ChatGPT-4o, Gemini 2.0 Pro, and DeepSeek-V3 can provide largely accurate and reproducible patient-facing information on CWD, with ChatGPT-4o showing the most favorable overall profile. However, important limitations remain, particularly in treatment-focused domains and with respect to hallucinations and subtle inaccuracies. These findings support the use of LLMs as supplementary tools for patient education in CWD, rather than as replacements for physician counseling. Future research should explore integration of these models with guideline-constrained frameworks, multilingual evaluation, and real patient cohorts to better define their safe and effective role in thoracic surgery and pediatric deformity care.

## Figures and Tables

**Table 1:** Distribution of CWD Questions by Domain.

Domain	Question Numbers	Number of Ques. (*n*)	Description of Content
General Chest Wall Anomalies (CWA)	1–10	10	Definition, prevalence, etiology, hereditary patterns, scoliosis association, indications for specialist referral.
Pectus Excavatum—Overview & Presentation	11–20	10	Clinical definition, epidemiology, typical symptoms, physical signs, posture, demographic distribution.
Pectus Excavatum—Diagnosis	21–27	7	Role of lab testing, chest radiography, CT/Haller index, echocardiography, pulmonary function testing, differential diagnoses.
Pectus Excavatum—Treatment & Follow-up	28–41	14	Vacuum bell therapy, indications for MIRPE/Nuss, best age for surgery, activity restrictions, recurrence risk, postoperative care.
Pectus Carinatum—Overview & Presentation	42–51	10	Definition, types (chondrogladiolar/chondromanubrial), symptoms, complications, cardiopulmonary impact.
Pectus Carinatum—Diagnosis	52–57	6	Clinical examination, chest X-ray, CT/MRI, pressure assessment, pulmonary/cardiac testing.
Pectus Carinatum—Treatment & Follow-up	58–69	12	External compression bracing (mechanism, effectiveness), bracing schedules, PC surgery, recurrence, sports participation.
Quality-of-Life (QoL) Questions	70–80	11	Exercise tolerance, dyspnea, fatigue, psychosocial impact, body-image concerns, social functioning.
Total	1–80	80	—

**Table 2:** Overall Accuracy and Error Characteristics of the Three LLMs.

Metric	ChatGPT-4o	Gemini 2.0 Pro	DeepSeek-V3	*p*-Value *
	Mean ± SD or Median (IQR) or *n* (%)			
Accuracy score	1.51 ± 0.66	1.73 ± 0.78	1.88 ± 0.90	—
	1 (1–2)	2 (1–2)	2 (1–3)	0.002 *
Score = 1 (Fully accurate)	52 (65.0%)	44 (55.0%)	40 (50.0%)	—
Score ≥ 3 (Partially incorrect or incorrect)	10 (12.5%)	16 (20.0%)	22 (27.5%)	—
Hallucinations	4 (5.0%)	6 (7.5%)	9 (11.25%)	—

*: Friedman test for overall accuracy score comparison.

**Table 3:** Domain-Specific Median Accuracy Scores of the Three LLMs.

Domain	ChatGPT-4o	Gemini 2.0 Pro	DeepSeek-V3
General Chest Wall Anomalies (CWA)	1	1	2
Pectus Excavatum—Overview & Presentation	1	1	2
Pectus Excavatum—Diagnosis	1	2	2
Pectus Excavatum—Treatment & Follow-up	2	2	3
Pectus Carinatum—Overview & Presentation	1	1	2
Pectus Carinatum—Diagnosis	1	2	2
Pectus Carinatum—Treatment & Follow-up	2	2	3
Quality-of-Life (QoL)	1	1	2

**Table 4:** Pairwise Comparison of Accuracy Scores Between LLMs.

Comparison	Z Value	*p* (Uncorrected)	*p* Value *	Effect Size (r)
ChatGPT-4o vs. Gemini 2.0 Pro	−3.21	0.0013	0.004	0.36
ChatGPT-4o vs. DeepSeek-V3	−4.10	<0.001	<0.005	0.46
Gemini 2.0 Pro vs. DeepSeek-V3	−1.95	0.051	0.153	0.22

* Bonferroni test.

**Table 5:** Intra-Model Reproducibility of LLM Responses.

Model	*n*	Exact Agreement *n* (%)	Similar Agreement *n* (%)	Weighted κ (95% CI)
ChatGPT-4o	80	56 (70.0%)	72 (90.0%)	0.82 (0.76–0.88)
Gemini 2.0 Pro	80	52 (65.0%)	70 (87.5%)	0.78 (0.71–0.85)
DeepSeek-V3	80	50 (62.5%)	68 (85.0%)	0.76 (0.69–0.84)

**Table 6:** Inter-Rater Reliability for Accuracy Scoring.

Model/Overall	Fleiss’ κ	95% CI
ChatGPT-4o	0.72	0.66–0.78
Gemini 2.0 Pro	0.66	0.59–0.73
DeepSeek-V3	0.67	0.60–0.74
Overall (all models combined)	0.69	0.63–0.74

## Data Availability

The study did not involve human participants, clinical records, or identifiable data. All analyses were conducted using a synthetic dataset generated specifically for methodological evaluation and reproducibility testing. The full set of patient-facing questions used in the study is provided as Supplementary File S1. No additional raw data are available, as all outputs were generated directly by publicly accessible large language models (ChatGPT-4o, Gemini 2.0 Pro, and DeepSeek-V3) using newly created accounts without fine-tuning or external datasets. All procedures and prompts used for response generation are fully described in the Methods section to ensure complete reproducibility.

## Supplementary Materials

The following supporting information can be downloaded at https://www.mdpi.com/article/10.3390/diagnostics16040589/s1: Supplementary File S1. Complete List of the 80 Patient-Facing Questions on Chest Wall Deformities [4,52,53,54,55,56].
