# Supplementary material for: Performance of ChatGPT-4o, Gemini 2.0 Pro, and DeepSeek-V3 in Patient-Facing Information on Chest Wall Deformities: A Comparative Evaluation of Accuracy, RELIABILITY, and Reproducibility
Source: Diagnostics (Basel). 2026 Feb 15;16(4):589. doi: 10.3390/diagnostics16040589 (PMC12939082; doi:10.3390/diagnostics16040589)
Supplement: Supplementary file 1 [file diagnostics-16-00589-s001.zip › diagnostics-4110226-supplementary.pdf]

Supplementary File S1. Complete List of the 80 Patient-Facing Questions on Chest Wall Deformities

Chest Wall Anomalies

1. What are common chest wall anomalies? (1)
2. How common are chest wall anomalies? (1)
3. Do all CWA need to be repaired? (1)
4. Is fixing a CWA cosmetic procedure? (1)
5. What is the difference between pectus excavatum and pectus carinatum? (1)
6. What causes chest wall anomalies? (1)
7. Some areas of chest seem to be pushed out and others seem to be depressed. Is this pectus excavatum or a carinatum? (1)
8. Other members of my family also have chest wall anomalies. Is it common? (1)
9. My child has scoliosis as well as pectus. Is that common? (1)
10. At what age should a person with a CWA be evaluated by a surgeon with special expertise in these conditions? (1)

Pectus Excavatum

Overview and Presentation

11. What is pectus excavatum? (2)
12. What is the prevalance of pectus excavatum? (2)
13. What causes pectus excavatum? (2)
14. Is pectus excavatum hereditary? (3)
15. How does the prevalance of pectus excavatum vary by sex and race? (2)
16. At what age pectus excavatum typically identified? (2)
17. How does pectus excavatum affect pulmonary function? (2)
18. How is cardiac function affected by pectus excavatum? (2)
19. What are the physical signs of pectus excavatum? (2)
20. What is pectus posture in pectus excavatum? (2)

Diagnosis

21. What is the role of lab testing in the diagnosis of pectus excavatum? (2)
22. What is the role of imaging studies in the diagnosis of pectus excavatum? (2)
23. What is the role of chest radiography in the diagnosis of pectus excavatum? (2)
24. What is the role of CT scanning in the diagnosis of pectus excavatum? (2)
25. What is the role of echocardiography in the diagnosis of pectus excavatum? (2)
26. What are the roles of pulmonary function and cardiology tests in the workup of pectus excavatum? (2)
27. What are the differential diagnoses for Pectus Excavatum? (2)

Treatment and Follow-up

28. Which specialist consultations are needed for the management of pectus excavatum? (2)
29. What are the treatment options for pectus excavatum? (2)
30. Is there a way to correct pectus excavatum without surgery? (1)
31. Will correcting pectus excavatum resolve the associated medical problems? (1)
32. What are the indications for surgical repair for pectus excavatum? (2)

33. What is the surgical procedure for minimally invasive repair of pectus excavatum (MIRPE)? (2)
34. What is the best age for surgical repair for pectus excavatum? (1)
35. What are the activity restrictions following surgical repair of pectus excavatum? (2)
36. Is physical exercise recommended for an adult affected by pectus excavatum? (3)
37. What are the risks and benefits of minimally invasive repair of pectus excavatum (MIRPE)? (2)
38. What is the mortality risk of minimally invasive repair of pectus excavatum (MIRPE)? (2)
39. What kind of activities are most advisable for pectus excavatum? (3)
40. What activities should be avoided by pectus excavatum patients? (3)
41. Can the excavatum come back after surgical correction? (1)

Pectus Carinatum

Overview

42. What is a pectus carinatum? (4)
43. How common is it? (5) *(6)
44. What causes pectus carinatum? (5)
45. What are the types of pectus carinatum?*(6)
46. What are the symptoms of pectus carinatum?*(6)
47. Is pectus carinatum dangerous? *(6)
48. What medical problems are caused by pectus carinatum? (1)
49. How does pectus carinatum affect pulmonary function? (2)
50. How is cardiac function affected by pectus carinatum? *(6)
51. What are the complications of pectus carinatum? *(6)

Diagnosis

52. What are the signs and symptoms? (5)
53. How is pectus carinatum diagnosed? (5)(6)
54. What tests will be done to diagnose pectus carinatum? (6)
55. What are the roles of pulmonary function and cardiology tests in the workup of pectus carinatum?(7)
56. How do we determine the severity of pectus carinatum? (7)
57. What are the other tests used in diagnosis? (7)

Treatment And Follow Up

58. How is a pectus carinatum treated? (4)
59. Is surgery necessary to correct pectus carinatum? (1)
60. What is the right age to correct pectus carinatum? (1)
61. Can the pectus carinatum comeback after correction? (1)
62. What is external compressive bracing for pectus carinatum? (8)
63. How does bracing for pectus carinatum work? (9)
64. Can the skin be affected from pectus brace? (8)
65. How long will my child have to wear a chest brace for pectus carinatum? (9)
66. Is pectus carinatum brace visible under my clothes? (8)
67. What are the risks and benefits of using a brace for pectus carinatum? (9)
68. Can I still play sports after bracing for pectus carinatum? (8)
69. Do I need a course of physiotherapy for pectus carinatum? (8)

Quality Of Life Questions

70. Will I have difficulty playing/doing exercise because of my chest deformity? (11)
71. Will I ever feel shortness of breath/respiratory difficulty due to my chest deformity? (11)
72. Could my chest deformity cause me to feel tired? (11)
73. How does chest wall anomalies affect self-image and body image in young individuals? (12)
74. In what ways are children with pectus carinatum disadvantaged compared to their peers? (12)
75. In what ways are children with pectus excavatum disadvantaged compared to their peers? (12)
76. What are the effects of pectus carinatum has on my child's life? (12)
77. What are the effects of pectus excavatum has on my child's life? (12)
78. Are there specific activities that children and adolescents with pectus carinatum find challenging? (11)
79. Are there specific activities that children and adolescents with pectus excavatum find challenging? (11)
79. What impact does the treatment plan for pectus carinatum have on my child's quality of life? (12)
80. What impact does the treatment plan for pectus excavatum have on my child's quality of life? (12)

References

(1) https://www.pectustreatment.com/faq
(2) https://emedicine.medscape.com/article/1004953-overview
(3) https://pectusup.com/en/frequently-asked-question/
(4) https://www.childrenshospital.org/sites/default/files/2022-05/surgery-fact-sheet-pectus-carinatum-english.pdf
(5) https://www.asthmaandlung.org.uk/conditions/pectus-carinatum-pigeon-chest-causes-symptoms-treatment
(6) https://my.clevelandclinic.org/health/diseases/15790-pectus-carinatum
(7) https://emedicine.medscape.com/article/1003047-workup
(8) https://www.pectusclinic.com/treatments/bracing/
(9) https://www.webmd.com/children/what-to-know-about-bracing-pectus-carinatum
(10) https://newsnetwork.mayoclinic.org/discussion/mayo-clinic-q-and-a-pediatric-pectus-excavatum/
(11) Lawson ML, Cash TF, Akers R, Vasser E, Burke B, Tabangin M, Welch C, Croitoru DP, Goretsky MJ, Nuss D, Kelly RE Jr. A pilot study of the impact of surgical repair on disease-specific quality of life among patients with pectus excavatum. J Pediatr Surg. 2003 Jun;38(6):916-8. doi: 10.1016/s0022-3468(03)00123-4. PMID: 12778393.
(12) Paulson JF, Ellis K, Obermeyer RJ, Kuhn MA, Frantz FW, McGuire MM, Ortiz E, Kelly RE Jr. Development and validation of the Pectus Carinatum Body Image Quality of Life (PeCBI-QOL) questionnaire. J Pediatr Surg. 2019 Nov;54(11):2257-2260. doi: 10.1016/j.jpedsurg.2019.03.019. Epub 2019 Apr 24. PMID: 31056346.
